# Patient-Reported Outcome and Experience Measures (PROM/PREM) in Patients Undergoing Liver Surgery with Enhanced Recovery after Surgery (ERAS^®^): An Exploratory Study

**DOI:** 10.3390/healthcare12060629

**Published:** 2024-03-11

**Authors:** Daniela Rappold, Stefan Stättner, Elisabeth Nöhammer

**Affiliations:** 1 Department of General, Visceral and Vascular Surgery, Oberösterreichische Gesundheitsholding, 4840 Vöcklabruck, Austria; s.staettner@icloud.com; 2 Department of Public Health, Health Services Research & HTA, UMIT TIROL—Private University for Health Sciences and Health Technology, 6060 Hall in Tirol, Austria

**Keywords:** enhanced recovery after surgery, ERAS, PROM, PREM, liver surgery

## Abstract

Background: ERAS^®^ (Enhanced Recovery after Surgery) is an evidence-based multidisciplinary approach focusing on optimizing outcomes after surgery through structured clinical pathways. This study aimed to assess patient-reported outcome and experience measures (PROM/PREM), which are not routinely assessed after liver surgery within an ERAS^®^ protocol. Methods: Routine outcome parameters were extracted from clinical documentation. Using qualitative content analysis, PROM and PREM were retrospectively identified in 13 case records. In a prospective survey of 10 participants, PROM was assessed at three timepoints using the EQ-5D-5L questionnaire. PREM were collected at discharge. Results: The following PROM categories occurred in the retrospective content analysis: *appetite* (84.6%), *pain/discomfort* (76.9%), *mobility* (69.2%), *wound condition* (69.2%), and *weight* (61.5%). The categories of *continuity of care* (92.0%) and *information*, *communication*, *education* (69.0%) emerged as PREM. Descriptive changes in health state were shown for all EQ-5D-5L dimensions and timepoints. At discharge, *mobility*, *selfcare*, *usual activities*, and *pain/discomfort* tended to be worse, whereas *anxiety/depression* decreased gradually from preoperatively to the 4 week follow-up. There was high satisfaction with interprofessional care services and experienced cooperation between professionals. Conclusions: PROM and PREM are helpful to incorporate patients’ perspectives after liver surgery within an ERAS^®^ pathway and should be collected routinely in clinical practice.

## 1. Introduction

ERAS^®^ (Enhanced Recovery after Surgery) is an evidence-based multidisciplinary and patient-centered treatment concept focusing on optimizing outcomes after surgery through the implementation of clinical pathways [1,2]. These include detailed patient preparation and information before surgery and a structured follow-up, both by specialized healthcare staff. Improved outcomes through ERAS^®^ have been documented primarily for parameters like reduced length of stay (LOS), complications and readmissions [3].

This optimization is achieved through a multidimensional approach (e.g., preoperative education and patient optimization, minimal-invasive surgery, multimodal opioid-sparing analgesia and fluid management, early oral nutrition, early mobilization, continuous audit) carried out by a multiprofessional team (surgeons, anesthetists, dieticians, physiotherapists, nurses) [1,2] with an ERAS nurse who is responsible for preoperative patient education [4], perioperative counselling during patient recovery [5], data collection [5] and post-discharge follow-up [6].

However, outcome evaluation in ERAS^®^ so far mainly focuses on outcomes like LOS and postoperative complications, as well as physiological recovery after surgery in the hospital and 30 days after surgery [3,7].

The present consensus recommendations emphasize the incorporation of quality-of-life parameters, such as patient-reported outcome measures (PROMs) and patient-reported experience measures (PREMs), when evaluating the quality of surgical interventions to ensure this is covered from the perspective of all stakeholders [8]. PROM refers to patients’ self-reported health state, such as physical symptoms and functioning, and thus provides information about the impact of a disease or treatment on patients [9,10]. PREM, on the other hand, refer to patients’ experiences concerning their interactions with the healthcare system and the quality of care provided [10].

PROM and PREM are not routinely collected as part of the ERAS^®^ audit [7,11] but are becoming increasingly important regarding patient-centeredness and quality of life research, e.g., in (abdominal) surgery [8,12] and in terms of a multidimensional understanding of the recovery process [13]. Moreover, (ERAS) nurse-led telephone follow-up in the ERAS^®^ pathway can be used to evaluate outcomes such as pain, nutrition and mobility after discharge [6,14], and also provide insight into the patient’s experience in terms of early discharge [14].

The internationally recognized ERAS^®^ Society has issued consensus guidelines for various surgical domains [5], including liver surgery, with guidelines first published in 2016 [15] and updated in 2022 [16]. The benefits of the ERAS^®^ pathway for liver surgery have been well described in the literature, particularly in terms of classical outcome parameters like reduced LOS, treatment costs, perioperative complications and readmissions [17,18,19]. Despite scientific evidence regarding enhanced outcomes when implementing the ERAS^®^ pathway in clinical practice [17,18,19], there was no ERAS^®^-certified center in Austria until September 2023 [20].

Thus, an exploratory (pilot) study is conducted to evaluate the effects of the ERAS^®^ approach on PROM and PREM in the first Austrian ERAS^®^-certified hospital. There, the current focus is on liver surgery.

The following research questions are to be answered using a mixed-methods approach:

(1) Which PROM and PREM can be derived from the post-discharge follow-up telephone calls conducted by the ERAS nurse after liver surgery within the ERAS^®^ pathway?
(2) How does the health status, as evaluated by EQ-5D-5L, change from preoperatively to discharge and then four weeks postoperatively?
(3) How do patients experience the care provided while undergoing liver surgery in the hospital, specifically in relation to interprofessional collaboration and service integration within the ERAS^®^ pathway?

## 2. Materials and Methods

The pilot study reported here was conducted in an Austrian hospital using a mixed-methods approach consisting of a retrospective content analysis according to Mayring [21] and a prospective questionnaire survey.

Despite the lack of a comparative cohort in the setting investigated due to the novelty of the ERAS^®^ pathway in general and for liver surgery in particular, the selected mixed methods approach allows for the creation of a more comprehensive database for evaluating classic outcome parameters in connection with patient-reported outcomes and experiences. For a conclusive linking interpretation, the same inclusion and exclusion criteria were applied to both cohorts (see Appendix A).

A positive ethics vote has been obtained (see Institutional Review Board Statement).

### 2.1. Qualitative Content Analysis

Qualitative content analysis is based on the documentation of the standardized follow-up calls after surgery and discharge. It focuses on the patients’ self-reported health status and the patient’s experiences. After the definition of the material (the documentation of standardized follow-up calls with patients who had undergone liver surgery from July 2022 to December 2022), a deductive coding guide was designed (see Section 2.1.1). This was open for inductive amplifications to cover all PROM/PREM aspects raised by the patients. Coding rules were applied, and anchor examples were chosen. The analysis was conducted using the Software QCA map (V.1.2.0) (https://www.qcamap.org/ui/de/home, accessed on 9 March 2024). The full coding guide (German) is available on request from the authors.

The follow-up telephone calls of 13 participant cases that met the inclusion and exclusion criteria (see Appendix A) during the period of interest were systematized and given a consecutive number (FU_1 to FU_13) to enable the anchor examples to be referenced.

Microsoft Excel was used to present the quantitative distribution of the identified deductive and inductive categories.

#### 2.1.1. Deductive Analysis

Five subcategories were defined as equivalent to the five dimensions of the EQ-5D-5L (see Section 2.2.1) with a three-level ordinal category scheme (no–moderate–severe problems or complaints).

As the aim of the article was not to generate new PROM [22,23], the further categories (subcategories) were derived from existing literature and comprise the surgery-relevant PROM nutrition (appetite*, weight*, *nausea*°) [14,24], excretion (vomiting*, urinary passage*, stools*) [6,24,25], sleep (sleeping behavior*, *fatigue*°) [14,24], and wounds (*wound condition*°) [6,14,24,25] with a rating scheme depending on the respective context (*reduced–equal–increased/°normal–divergent)

#### 2.1.2. Inductive Analysis

Inductive category formation was used to systematically record relevant uncoded spaces from the text material. These are PROM/PREM related aspects highlighted by the patients that were not represented by the deductive categories. These inductively created categories emerge from the recurring patterns or common themes that can be recognized in the text [21]. The aim was to summarize the identified text passages into new categories (analogous to the deductive categories of pain, nausea, etc.) through the process of abstraction. For PREM identification, the PREM categories in the NHS patient experience framework [26] (e.g., *information*, *communication*, *and education*; *access to care*) served as points of reference.

### 2.2. Prospective Questionnaire Survey

The prospective survey used the validated PROM questionnaire EQ-5D-5L [27], as well as PREM items on patient experience of care and service integration generated or adopted from the literature [28,29].

#### 2.2.1. EQ-5D-5L

The two-page EQ-5D-5L is a validated generic patient-reported outcome measure. The instrument consists of a descriptive system (EQ-5D Health Profile) covering five dimensions (mobility, self-care, usual activities, pain/discomfort, anxiety/depression) using five levels (no–slight–moderate–severe–extreme problems or complaints), with the best health state characterized by the code 11111 (indicating no problems/no complaints in any of the five dimensions) and the code 55555 describing the worst health state.

Moreover, it includes a thermometer-like visual analog scale (EQ VAS), on which health is rated from 0 to 100 (worst to best). Both elements allow a simple representation of the person’s current self-reported health state [27,30].

The use of the EQ-5D-5L for research purposes has been approved (see Data Availability Statement).

#### 2.2.2. PREM Survey

The PREM survey covers interprofessional care services and interprofessional collaboration and was designed in accordance with the research questions of the present study based on available English-language PREM questionnaires (HowRwe and Service Integration [28,29]).

The questionnaire consists of five items of interprofessional care services (*friendliness*; *listening and explaining*; *waiting times*; *appointment coordination*; *feeling informed*) that are rated on a four-point scale (very satisfied, satisfied, dissatisfied, very dissatisfied). Moreover, four dimensions of the perceived interprofessional collaboration (*Professionals talk to each other*; *Staff know what others are doing*; *I don’t have to repeat my story*; *Different professionals work well together*) are also rated based on four options (strongly agree–agree–neutral–disagree).

A text field for open comments was added to enable individual feedback (“If there is anything else you would like to tell us, please use the space provided here.”).

#### 2.2.3. Measurement Timepoints

In the context of a longitudinal survey, the EQ-5D-5L questionnaire was administered at three timepoints:

1. Preoperatively *(EQ5D5L-0)*
2. Postoperatively at discharge *(EQ5D5L-D)*
3. Four weeks postoperatively *(EQ5D5L-4W)*

The PREM questionnaire was handed out once on the day of discharge, as that is when it is best remembered.

#### 2.2.4. Analysis

Statistical analysis is carried out using IBM SPSS Statistics 29.0.0.0 after pseudonymization (internal coding with a consecutive number). Tables and diagrams are created in Microsoft^®^ Excel^®^ for Microsoft 365 MSO (Version 2402). Based on the exploratory study design and the resulting small sample size, descriptive statistics (minimum, median, maximum, mean, standard deviation) are used to present the results.

To facilitate statistical analysis and interpretation for the PREM questionnaire, the best answer option (very satisfied/strongly agree) is coded as “1” and the worst answer option (very dissatisfied/disagree) as “4” in SPSS or Excel.

In addition to PROM and PREM, classical outcomes like length of stay, complications, and readmission rates are of interest. The length of stay is defined as the time between surgery and discharge from the hospital in days. For the evaluation of complications, the Clavien Dindo classification (CD) [31] was used. Readmission is defined as rehospitalization within 30 days that is related to the primary surgery (for example, due to a complication related to the operation).

#### 2.2.5. Study Population

Of 14 people who fulfilled the inclusion criteria (see Appendix A) during the 6-month pilot-study period of (April 2023–September 2023), 11 out of a total of 14 possible cases consented to participate in the study. Two patients refused to participate in the study after being informed. Another case could not be informed about the study due to a language barrier and was therefore also excluded. One case from the eleven people mentioned above was excluded from the analysis after consent was given and the preoperative EQ-5D-5L questionnaire was completed due to death during the hospitalization and the resulting incomplete data.

Hence, complete data sets are available from ten patients who met the inclusion criteria and agreed to participate based on informed consent.

## 3. Results

This section presents the study population and the results of the qualitative and quantitative methodical steps.

### 3.1. Population Characteristics and Classical Outcomes

Population characteristics for retrospective content analysis (N = 13) and prospective questionnaire surveys (N = 10) are shown in Table 1. Patients in the retrospective analysis are on average seven years younger than those in the prospective questionnaire survey (55.5 years and 62.8 years, respectively).

Regarding the distribution of surgical approaches, it can be concluded that the proportion of minimally invasive procedures (robotic or laparoscopic) is comparable in both cohorts, at 84.7% in the retrospective analysis and 90% in the prospective analysis.

Both cohorts predominantly comprise patients with oncological diagnoses (76.9% and 70%), with the main diagnosis being colorectal liver metastasis and hepatocellular carcinoma.

Regarding classical outcomes (see Table 2), the median length of stay in the prospective cohort (five days) is lower than in the retrospective analysis (six days). In terms of readmission and complications, the two cohorts are comparable, with one readmission each within 30 days. While over 60% of the patients in both cohorts did not have any complications, 15.4% in the retrospective analysis and 10% in the prospective survey showed CD IIIa complications (requiring surgical, endoscopic or radiological intervention not under general anesthesia [31]).

### 3.2. Qualitative Content Analysis

#### 3.2.1. Deductive Qualitative Content Analysis

Thirteen follow-up telephone calls by the ERAS nurse with patients who had undergone liver surgery were conducted from July to December 2022.

The follow-up telephone calls included took place on the fifth day after discharge on average and lasted an average of 9 min (±4 min). These were documented with an average word count of 101, with a range of 38 to 184 words.

These documents were retrospectively analyzed using a coding guide (see above) consisting of the five EQ-5D-5L dimensions (mobility, self-care, usual activities, pain/discomfort, anxiety/depression) plus nutrition, excretion, sleep, and wounds as further deductively created categories. Most of these are asked for as a standard in the follow-ups and rated, which is why the descriptions, scores and differences could be analyzed. Some of the predefined outcomes were not identified in all follow-up telephone calls. The excretion-related outcome *urinary passage* was not identified in any, and the EQ-5D-5L dimension *self-care* was coded in only two follow-up telephone calls. In contrast, the EQ-5D-5L dimensions of *mobility* and *pain/discomfort* were coded in 69.2% and 76.9% of the documents, respectively.

The most frequently identified outcome is the nutrition-related PROM *appetite*, which is localized in eleven of the 13 follow-ups (corresponding to 84.6%). A constant appetite can be derived from three follow-ups (“*Reports good appetite*, *eats several small portions*”, FU_8), whereas four documents each show a reduced (“*Still restrained appetite*, *eats rather small meals*”, FU_11) or increased appetite (“*Good appetite*, *eats more than in hospital*”, FU_4) compared to preoperative or the time of discharge.

The outcomes *weight* and *wound condition* were also present in over 60% of the documents analyzed and assigned to the categories *nutrition* and *wounds*, respectively.

#### 3.2.2. Inductive Qualitative Content Analysis

The categories *coordination and continuity* *of care* (92%) and *information*, *communication and education* (69%) were discovered and categorized as PREM in most follow-up call documentations.

*Coordination and continuity of care* includes appointments at the general practitioner (“*Today at the GP for the removal of staples*, *remaining 2 staples will be removed by the GP on the day after*”, FU_2) or further appointments in the hospital (“*Appointment for hospital admission for planning of the chemotherapy is communicated*”, FU_2).

The PREM category of *information, communication and education* comprises two subcategories. The subcategory education by the ERAS Nurse (“*Instructed the patient to leave the wounds open from now on*”, FU_10) is derived from passages of four documents, and the subcategory information and communication by the ERAS Nurse is derived from reports in 8 out of 13 documents (“*Patient was told that she is welcome to contact me* [ERAS Nurse] *by telephone if she has any further questions*”, FU_9).

### 3.3. Prospective Quantitative Analysis

The results concerning the EQ-5D-5L and the changes in EQ VAS over the three timepoints presented in the following paragraphs are based on slightly different study populations. Data is available from all study participants at preoperative baseline (N = 10) and in the four-week follow-up (N = 10). However, data is available from only nine study participants for the survey time at the day of discharge (N = 9), as one participant had not been discharged at the measurement time point four weeks postoperatively.

The preoperative survey was conducted on average six days before surgery. The second survey date is the day of discharge, and the four-week follow-up was carried out on average on postoperative day 28.

#### 3.3.1. EQ-5D-5L Changes over the Three Survey Timepoints

**Figure 1:**
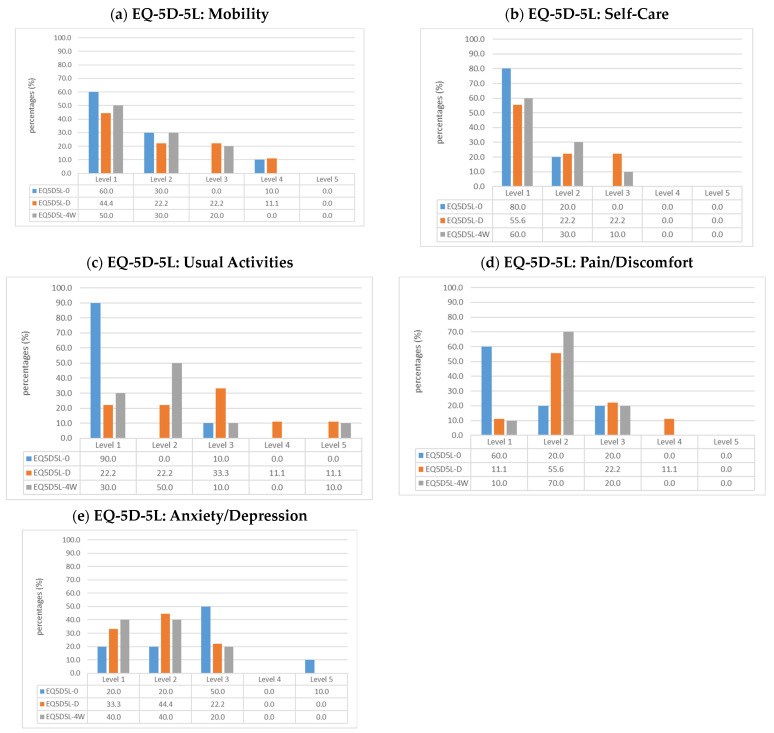
Changes of EQ-5D-5L levels over the three survey timepoints in the five EQ-5D-5L dimensions as percentage [27]; (**a**) Mobility; (**b**) Self-Care; (**c**) Usual Activities (e.g., work, study, housework, family or leisure activities); (**d**) Pain/Discomfort; (**e**) Anxiety/Depression; Legend: EQ5D5L-0 = preoperatively, N = 10; EQ5D5L-D = at discharge, N = 9; EQ5D5L-4W = four-week follow-up, N = 10).

When comparing the EQ-5D-5L health profile before surgery with the profile at discharge, an initial decline in overall health, particularly for the EQ-5D-5L dimensions *mobility*, *self-care*, *usual activities*, and *pain/discomfort*, is visible (Figure 1a–d). In particular, the descriptive analysis regarding dimensions of *usual activities and pain/discomfort* at the time of discharge shows a shift to higher levels of issues compared to the preoperative status. This can be deduced from the selected higher levels (levels 3–5) in the dimensions mentioned. In the four-week follow-up, however, a shift to the lower levels (level 1 or 2) can be observed, which in turn indicates a better self-assessment of the general health status in the respective dimensions of the EQ-5D-5L. Despite lower values at the four-week follow-up, preoperative levels (modal value 1, “*no problems*”) are not reached. While 60% stated “*no pain or discomfort*” before surgery, level 1 was only selected once at discharge, and once at the four-week follow-up surgery. At these two points in time, most of the assessments were at level 2 (“*I have slight pain or discomfort*”).

Regarding the EQ-5D-5L dimension *mobility* over the course of the three measurement points, 90% of patients state no or slight problems before surgery, whereas on the day of discharge, only two-thirds (66.7%) state no or slight problems (Level 1 or 2). In the four-week follow-up, 80% describe no or slight problems, and only two patients (20%) express moderate problems walking around. None of the patients selected level 5 (“I am unable to walk”) in relation to mobility at any of the three measurement points.

In contrast, the situation is different regarding the fifth EQ-5D-5L dimension of *anxiety/depression* (Figure 1e). While half of the patients stated that they were “*moderately anxious or depressed*” (Level 3) at the time of the preoperative survey, there was already a shift towards levels 1 and 2 at the time of discharge (“*I am not/I am slightly anxious or depressed*”).

#### 3.3.2. EQ VAS

At all three timepoints, the minimum on the EQ VAS reported by the patients was 50, with a maximum of 95 (preoperatively, day of discharge) and 90 (4 weeks postoperatively).

**Figure 2:**
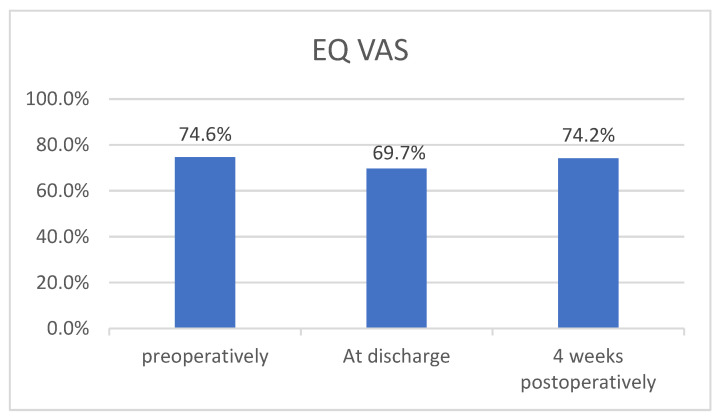
Change of EQ VAS for the three survey timepoints (mean values, percentages).

Figure 2 shows the mean values in percentages based on the responses of 10 study participants for baseline and four-week follow-up and 9 participants at discharge.

This indicates a slight decrease in the mean EQ VAS from baseline (74.6%) to discharge (69.7%), with a subsequent increase to 74.2% at four-week follow-up.

A possible trend resulting from the descriptive analysis of the EQ VAS data at the three survey time points based on the calculated mean values (74.6% vs. 69.7% vs. 74.2%) has no statistical significance due to the limited sample size.

Nevertheless, the longitudinal trend shows that 70% of patients reported the same or higher EQ VAS scores at the four-week follow-up compared to their preoperative EQ VAS scores.

#### 3.3.3. PREM

Table 3 shows the mean values of the answers of 10 patients who completed the PREM survey at discharge.

All patients are “very satisfied” with the *friendliness* of the interprofessional team, with the mean value being the best rating of 1.00. Nine out of ten patients fully agree with the statement “*Different professionals work well together*,” resulting in a mean of 1.10. As shown in Table 3, the other aspects of interprofessional care services and collaboration are also experienced as very positive.

The free text field was only used by one study participant concerning a comment on *waiting times*, the item with the lowest mean rating (1.40) in descriptive analysis.

## 4. Discussion

This exploratory mixed-methods study provides insight into PROM and PREM relevant for patients undergoing liver surgery following the ERAS^®^ pathway.

Both cohorts show comparable outcomes in terms of LOS, complications, and readmissions, with most patients having malignant liver diseases. Nevertheless, a slight trend of the effect of structured treatment according to the ERAS^®^ pathway on reduction in LOS as described in the literature [17,19] is already visible. The median length of stay in the two cohorts differs by one day (6 days in retrospective and 5 days in prospective analysis). However, no actual pre-ERAS^®^ data from the hospital in question and no ERAS^®^-reference data are available since there is no comparable ERAS^®^-certified center in Austria.

Liver surgery is associated with an increased risk of postoperative complications, especially in terms of oncological surgery [32]. In the present study, complications are assessed through the Clavien-Dindo classification, resulting in low serious complications in both cohorts (15.4% in the retrospective analysis and 10% in the prospective survey showed Clavien-Dindo IIIa complications). Although well-established, the classification is controversial in the literature since many studies focus on severe complications, resulting in an underrepresentation of less serious complications [33]. Alternatively, the comprehensive complication index (CCI) could be used [8], as the CCI considers the patient’s perspective by assigning weights to complications [33].

### 4.1. Qualitative Data

In the case of intended early discharge according to the ERAS^®^ treatment pathway, patients should be informed about the planned early discharge before surgery [34]. Moreover, telemedical monitoring should be offered, for example, through structured telephone follow-up after discharge, since the period around discharge and the first time at home is considered vulnerable [24,34]. Structured follow-up calls conducted by a professional (e.g., an ERAS Nurse) [6,24] can help to ensure early discharge and address the patient´s need for ongoing telemedical care the first time after discharge [34,35]. Moreover, these calls can provide vital information about the quality of care and outcomes of treatment.

The follow-up calls for this study were conducted on the fifth day after discharge and lasted 9 min on average, which corresponds to the duration described in the literature for colorectal surgery (10 to 17 min) [14,36]. Patients generally welcome follow-up telephone calls and are reassured about their recovery through the conversation with the specialist [14,35], which also became evident in the present exploratory qualitative content analysis. In addition, our data indicates that nutrition-, wound-, sleep-, and excretion-related PROM can mostly be derived from the follow-up telephone calls carried out by the ERAS nurse. Concerning PREM, the retrospective analysis of the follow-ups identifies outcomes that refer to continuity of care and education, plus information and communication during treatment. As a result of the inductive category formation, the PREM categories [26] *coordination and continuity of care*, as well as *information*, *communication*, *and education*, were identified in most of the analyzed documents, implying that the required continuity of care can be considered fulfilled. Regarding the results of the PREM questionnaire, patients described interprofessional care services and collaboration during their hospital treatment as very positive.

### 4.2. EQ-5D-5L

In the prospective questionnaire survey, changes in general health status assessed by EQ-5D-5L within the perioperative treatment period are revealed. A deterioration in the EQ-5D-5L dimensions of *mobility*, *self-care*, *usual activities*, and *pain/discomfort* at the time of discharge, is shown, which then diminishes in the four-week follow-up. The mean values of the EQ VAS also show a deterioration in the state of health at the time of discharge with a positive trend towards recovery in the four-week follow-up. Regarding the EQ-5D-5L dimension of *anxiety/depression*, most patients stated that they were “moderately anxious or depressed” before surgery, which improved postoperatively.

Preoperative anxiety and cognitive overload are also described in the literature [37], since patients receive information about the surgery, possible complications, the potential cancer diagnosis, and the ERAS^®^ pathway simultaneously [37]. Considering that preoperative anxiety and low self-efficacy can have a negative impact on post-surgery outcomes, addressing the issue through talks with the treatment team or by offering cognitive behavioral therapy [37] as part of the prehabilitation program before surgery [38] could be beneficial.

In combination with the qualitative data, the results indicate that in addition to generic patient-reported outcomes assessed through EQ-5D-5L, disease- or condition-specific outcomes are important for patients during their recovery, which is particularly evident in the post-discharge follow-up interviews conducted by the ERAS nurse. For example, nutrition-related outcomes like *appetite* and *weight* emerge as important PROMs in the qualitative content analysis, which is consistent with the findings of other literature on outcome assessment after surgery with ERAS^®^ [35].

These outcomes are not addressed in the EQ-5D-5L, which is a generic tool to assess health status in terms of health-related quality of life. Given that most patients included in both cohorts are cancer patients, a disease-specific PROM tool like the EORTC-QLQ-C30 [39] could be used additionally. This cancer-specific survey covers a total of 30 items, as well as nutrition-related outcome parameters like nausea, weight and vomiting [39]. Nevertheless, the EQ-5D-5L impresses through its shortness and generalizability and allows cross-population statements on the state of health [40,41].

### 4.3. Limitations

This study has several limitations. Due to stringent inclusion and exclusion criteria, both cohorts consist of small samples with (N = 13) in the retrospective qualitative content analysis and (N = 10) in the quantitative prospective questionnaire survey. Patients with multiple surgeries at one time or non-elective surgeries were excluded to facilitate replication.

While comparisons between groups can already be conducted with smaller samples, an N of at least 25–30 is recommended [42,43]. Depending on the planned analyses and parameters set, the requirements can also be much higher [44]. Therefore, the data collection is continuing. In the long run, cooperation with other hospitals regarding data collection might be possible as well.

Concerning the measurement timepoints of the EQ-5D-5L and EQ VAS, preoperative and four-week follow-ups are available for all patients. For one patient, however, data for regular discharge could not be analyzed with the others as this person was discharged weeks after the surgery.

The primary focus of the present pilot study was to show changes in the five dimensions of the EQ-5D-5L over the three timepoints. Intra-subject changes are not shown to respect data privacy in this small sample size.

Though the follow-up calls were standardized, they did not include scales. Therefore, their analysis was performed qualitatively and focused on identifying PROM and PREM. Due to the detailed documentation, the categorization scheme for the deductive analysis could be subdivided to cover three levels of problems/complaints (no–moderate–severe) based on the EQ-5D-3L, a previous version of the EQ-5D-5L [41]. As the latter uses five levels, a direct comparison between the retrospective qualitative (three levels) and the prospective quantitative data (five levels) concerning the EQ-5D dimensions is not feasible.

For further research, the EQ-5D-5L dimensions of self-care and usual activities should be routinely evaluated in the follow-up calls.

Lastly, social desirability must be mentioned since the ERAS nurse, as a person of trust for the patients, was also responsible for conducting the study in accordance with her training. While this allows for richer data as patients report more to ensure personalized care, some might be afraid to voice direct criticism. This danger was reduced by structured follow-up calls with specific questions regarding issues and by providing surveys not only before but also after treatment at discharge and outpatient follow-up appointments. The standardization of the calls also serves to safeguard the reliability of the data.

### 4.4. Strengths

The strength of the study design lies in its exploratory nature combined with a mixed-methods design and longitudinal data. Long-term data assessed through the EQ-5D-5L four weeks postoperatively provides an initial insight into the course of the patient’s recovery in terms of their self-reported health status.

Moreover, the study is innovative as it investigates the not yet widely implemented ERAS^®^ treatment concept for liver surgery within the German-speaking part of Europe and combines it with PROM and PREM.

The mixed-methods approach and the integration of retrospective and prospective data enable a more comprehensive perspective on PROM and PREM for liver surgery, according to ERAS^®^.

## 5. Conclusions

Standardized use of PROM and PREM in clinical practice enables quantitative measurement and continuous improvement of health outcomes in surgery [8] while promoting patient-centered healthcare [10]. To ensure a comprehensive evaluation of outcomes after surgery according to the ERAS^®^ treatment pathway, PROM and PREM should be routinely collected at predefined timepoints to evaluate changes in the health-related quality of life of patients undergoing liver surgery.

The findings from this exploratory study can serve as the basis for generating hypotheses for future research.

Further studies using the EQ-5D-5L should also evaluate the intra-subject change in the health profiles over the survey period, e.g., by means of the Pareto Classification of Health Change (PCHC) [27,45].

In addition, we suggest including alternative outcome parameters, such as the CCI for complications, which also takes the patient’s perspective into account [8]. Further studies should also consider long-term patient-reported outcomes, recognizing that recovery is a multidimensional process that extends well beyond four weeks post-surgery [46] until the return to normal function [7]. Additionally, the duration of the latter could be examined more closely by also including family and relatives in the study, as they might evaluate patients’ progress more critically.

## Figures and Tables

**Table 1:** Population characteristics.

	Qualitative Content Analysis N = 13	Prospective Questionnaire Survey N = 10
**Age [years]**		
Mean ± SD	55.5 ± 10.0	62.8 ± 11.5
Median	56	65
**Sex**		
Male	46.2%	70.0%
Female	53.8%	30.0%
**Surgical approach**		
Robotic	61.5%	70.0%
Standard Laparoscopic	23.2%	20.0%
Open	15.3%	10.0%
**Main procedure name**		
Segmentectomies/Atypical resections	61.5%	80.0%
Wegde resections		10.0%
Extended left hemihepatectomy	15.4%	
Right hemihepatectomy	23.1%	
Extended right hemihepatectomy		10.0%
**Histological diagnosis**		
Colorectal liver metastasis	53.8%	30.0%
Non-colorectal/non-neuroendocrine liver metastases	7.7%	10.0%
Perihilar cholangiocarcinoma	7.7%	
Intrahepatic cholangiocellular carcinoma	7.7%	
Hepatocellular carcinoma		30.0%
Hepatocellular adenomas	15.4%	
Hemangioma		10.0%
Other benign disease	7.7%	20.0%
**Oncological diagnosis**		
Yes	76.9%	70.0%
No	23.1%	30.0%

SD = Standard deviation.

**Table 2:** Classical outcome parameters.

	Qualitative Content Analysis N = 13	Prospective Questionnaire Survey N = 10
**LOS ^1^ [days]**		
Mean ± SD	7.1 ± 6.0	8.8 ± 11.8
Median	6.0	5.0
**Complications [CD ^2^]**		
No complications	61.5%	60.0%
CD I	7.7%	0%
CD II	15.4%	30.0%
CD IIIa	15.4%	10.0%
**Readmission ^3^**	7.7%	10.0%

^1^ LOS = Length of stay after surgery ^2^ CD = Clavien-Dindo Classification of surgical complications [31] ^3^ Readmission within 30 days after surgery; SD = Standard deviation.

**Table 3:** PREM survey analysis.

	Mean (N = 10)
**Interprofessional care services ^1^**	
Friendliness	1.00
Listening and explaining	1.20
Waiting times	1.40
Appointment coordination	1.30
Feeling informed	1.30
**Interprofessional collaboration ^2^**	
Professionals talk to each other	1.20
Staff know what others are doing	1.20
I don´t have to repeat my story	1.30
Different professionals work well together	1.10

^1^ 1 = very satisfied, 2 = satisfied, 3 = dissatisfied, 4 = very dissatisfied. ^2^ 1 = strongly agree, 2 = agree, 3 = neutral, 4 = disagree.

## Data Availability

Data generated as part of this study are available from the corresponding author on reasonable request. EQ-5D-5L can be used free of charge for in clinic surveys; Non-commercial use of EQ-5D, category “Research (intend to publish the results); (EuroQuol Registration ID: 51541; August 2022–August 2024”.

## Appendix A. Inclusion and Exclusion Criteria

**Inclusion criteria** Adult men and women (≥18 years), who undergo liver surgery and are cared for by the interprofessional treatment team in accordance with the ERAS^®^ treatment pathway in one Austrian hospital.
Benign tumors - Hepatocellular adenomas - Hemangiomas - Focal Nodular hyperplasia Primary liver tumors - Hepatocellular carcinoma - Intrahepatic cholangiocellular carcinoma - Perihilar cholangiocarcinoma (Klatskin Tumor) Secondary liver tumors - Colorectal liver metastases - Neuroendocrine liver metastases - Non-colorectal/non-neuroendocrine liver metastases
Elective liver surgery - Resection of liver metastases - Wedge resection - Atypical resection - Left/right hemihepatectomy - Extended hemihepatectomy left/right - Mesohepatectomy
Surgical approach - Open - Minimally invasive (laparoscopic or robotic) - Converted from minimally invasive to open
**Exclusion criteria**
Combined surgery of multiple organs (e.g., surgery for primary rectal cancer and removal of colorectal liver metastases at the same time)
Additionally for part 1 (retrospective qualitative content analysis) - Missing data: no follow-up telephone call - Missing data: no postoperative care by ERAS Nurse
Additionally for part 2 (prospective questionnaire survey) - Lack of informed consent of the patients for the prospective study - Withdrawal of participation: If consent is withdrawn during the course of the study, the corresponding case is excluded from the data analysis
